# Recent advances and applications of CRISPR-Cas9 in cancer immunotherapy

**DOI:** 10.1186/s12943-023-01738-6

**Published:** 2023-02-16

**Authors:** Zaoqu Liu, Meixin Shi, Yuqing Ren, Hui Xu, Siyuan Weng, Wenjing Ning, Xiaoyong Ge, Long Liu, Chunguang Guo, Mengjie Duo, Lifeng Li, Jing Li, Xinwei Han

**Affiliations:** 1 Department of Interventional Radiology, The First Affiliated Hospital of Zhengzhou University, Zhengzhou, 450052 Henan China; 2 Interventional Institute of Zhengzhou University, Zhengzhou, 450052 Henan China; 3 Interventional Treatment and Clinical Research Center of Henan Province, Zhengzhou, 450052 Henan China; 4 Department of Respiratory and Critical Care Medicine, The First Affiliated Hospital of Zhengzhou University, Zhengzhou, 450052 Henan China; 5 Department of Emergency Center, Zhengzhou University People’s Hospital, Zhengzhou, 450003 Henan China; 6 Department of Hepatobiliary and Pancreatic Surgery, The First Affiliated Hospital of Zhengzhou University, Zhengzhou, 450052 Henan China; 7 Department of Endovascular Surgery, The First Affiliated Hospital of Zhengzhou University, Zhengzhou, 450052 Henan China; 8 Cancer Center, The First Affiliated Hospital of Zhengzhou University, Zhengzhou, 450052 Henan China

**Keywords:** CRISPR-Cas9, Cancer immunotherapy, Cellular therapy, NK/macrophage, Mechanism

## Abstract

The incidence and mortality of cancer are the major health issue worldwide. Apart from the treatments developed to date, the unsatisfactory therapeutic effects of cancers have not been addressed by broadening the toolbox. The advent of immunotherapy has ushered in a new era in the treatments of solid tumors, but remains limited and requires breaking adverse effects. Meanwhile, the development of advanced technologies can be further boosted by gene analysis and manipulation at the molecular level. The advent of cutting-edge genome editing technology, especially clustered regularly interspaced short palindromic repeats (CRISPR-Cas9), has demonstrated its potential to break the limits of immunotherapy in cancers. In this review, the mechanism of CRISPR-Cas9-mediated genome editing and a powerful CRISPR toolbox are introduced. Furthermore, we focus on reviewing the impact of CRISPR-induced double-strand breaks (DSBs) on cancer immunotherapy (knockout or knockin). Finally, we discuss the CRISPR-Cas9-based genome-wide screening for target identification, emphasis the potential of spatial CRISPR genomics, and present the comprehensive application and challenges in basic research, translational medicine and clinics of CRISPR-Cas9.

## Background

Surgery, radiotherapy, and chemotherapy [1–3] are recognized, preferred, and widely used worldwide for cancer therapies. Targeted therapy [4], photothermal and photodynamic therapies [5] are now improved and developed. However, individualized treatment of cancer is still in the initial stage. Radiation injury, drug toxicity [6–8], and other adverse reactions may occur in conventional curing and even lead to death [9], we urgently need to broaden new tools for cancer treatment. Cancer immunotherapy fights the growth and invasion of tumor cells by restoring or stimulating the immune system [10]. Immunotherapy generally includes cytokine therapy, immune checkpoint blockade, adoptive cellular immunotherapy (ACT), cancer vaccine, oncolytic virus therapy, DC cell therapy, and antibody-drug conjugate (ADC). Additionally, the anti-CD19 chimeric antigen receptor (CAR) demonstrated a breakthrough in curing liquid tumors [11]. In 2017, the anti-CD19 CAR was approved by the Food and Drug Administration (FDA) for treating refractory B-cell leukemia and lymphoma [12]. Regina et al. [13] reviewed advances in CAR-T therapise such as multiple approaches using synthetic biology and orthogonal receptors, aiming to overcome antigen escape and modulate the tumor microenvironment (TME). Nevertheless, several factors, such as the immunosuppressant antagonism and cytokine release syndrome (CRS) of chimeric antigen receptor T-cell (CAR-T), lead to immunotherapy being mostly limited to the experimental stage.

Generally, the development of sophisticated technology is associated with advances in gene analysis and manipulation at the molecular level [14]. In addition to the burgeoning field of base and prime editing, the efficiency and success of gene editing depends on whether double-strand breaks (DSBs) can be generated at specific, accurate, and predictable sites [15]. Consequently, broken ends can be rejoined through non-homologous end joining (NHEJ) and microhomology-mediated end joining (MMEJ), which may contribute to insertions and deletions (INDELs). Gene mutation can give rise to inactivation or be repaired by homologous recombination repair (HDR) pathway [16, 17]. We are endowed with four types of powerful nucleases, such as meganucleases, zinc-finger nucleases (ZFNs), transcription activator-like effector nucleases (TALENs) and clustered regularly interspaced short palindromic repeats (CRISPR)-associated protein 9 (CRISPR-Cas9) [18–20] (Table 1). Specifically, CRISPR-Cas9 is superior to the others in scalability, flexibility, and operability, which can be easily constructed by expressing different single guide RNAs (sgRNAs). The convenience of designing sgRNAs to target virtually any part of the genome - which enables the development of pooled, genome-scale CRISPR libraries (a feat that TALENs or ZFNs cannot achieve). Briefly, researchers have recognized CRISPR-Cas9 as the most promising gene editing approach [21].

**Table 1:** Comparison of the genome-editing tools we are endowed with

	Meganuclease	ZFN	TALEN	CRISPR-Cas9
Target site recognition	Protein-DNA interaction	Protein-DNA interaction	Protein-DNA interaction	RNA-DNA interaction
Target site	Single	Single	Single	Multiple
Efficiency	Low	Low	Low	High
Recognition	5′-TAGGGATAACAGGGTAAT-3′	Guanine-rich region	5′-T…….…..…..A-3′	……………5′-NGG-3′
Endonuclease	I-Crel/I-Scel (LAGLIDADG family)	FokI	FokI	Cas9
Design difficulty /Time cost	Complicated/Time-consuming	Complicated/Time-consuming	Complicated/Time-consuming	Simple/Time-saving
Other disadvantages	Potential genotoxicity/The length of the I-SceI site	Toxic/Expensive/Limiting target length is 3 multiples	Large protein size (difficult to deliver in the human genome)	Restricted by PAM/Off-target effects

## Basic mechanism of CRISPR-Cas9-mediated genome editing

Barrangou et al. [22] performed infection experiments with *Streptococcus thermophilus* and revealed that the CRISPR-Cas9 system provides resistance to phages, providing the first experimental evidence for its adaptive immunity role. Generally, CRISPR-Cas-mediated adaptive immunity occurs over three steps: acquisition, transcription, and interference [23]. It can be simply summarized as acquiring foreign DNA to integrate into the host CRISPR locus, producing mature crRNA (CRISPR-derived RNA) and Cas protein cleaving the target sequence under the guidance of RNA. In conclusion, The system includes a Cas9 nuclease and a guide RNA (gRNA). Generally, gRNA consists of crRNA and transactivating crRNA (tracrRNA) that forms base pairs with DNA target sequences, allowing Cas9 to introduce a site-specific DSB into DNA. TracrRNA:crRNA is usually designed as a single RNA complex (sgRNA). The two-component structure of sgRNA and Cas9 is easy to operate. Multiple editing of the target locus can be achieved by designing multiple sgRNAs. Collectively, the 5′ end recognizes a specific DNA target sequence by Watson-Crick base pairing, while the Cas9 nuclease binds to the 3′ end of the sgRNA, causing a target DSB at about 3 bp upstream of the protospacer adjacent motifs (PAMs) [24, 25]. By investigating the structure of *Streptococcus pyogenes* Cas9 [26], it was found that the mechanism of DSB may be implicated in the conformational change of RNA-target DNA binding. Of note, site-specific cleavage occurs at the location determined by base-pairing complementarity between the crRNA (sgRNA) and the target protospacer gene positions and short motifs (PAMs) juxtaposed to complementary regions of the target DNA [25]. In the absence of PAM, even entirely complementary target sequences cannot be recognized by Cas9 [27]. PAM is an important prerequisite for the design of sgRNAs. Of note, it has been demonstrated that PAM plays an indispensable role in the stages of adaptation and interference in type II systems [28]. After binding to the PAM and DNA-sgRNA hybrid formation, the RuvC and the HNH nuclease domain of Cas9 play significant roles in introducing DSBs into the target sequence [29]. Overall, the requirements for identifying target DNA are as follows: 1) site-specific complementarity between a 20-nucleotide crRNA sequence (sgRNA) and the target DNA; 2) an NGG protospacer motif adjacent to the target sequence (PAM) presence [26]. Subsequently, DSBs are repaired by the cellular self-repair mechanisms. NHEJ and MMEJ typically cause INDELs of genes to disrupt protein-coding sequences and develop functional knockouts. The repair template consists of the target gene and homologous sequences of the target sequence (homologs) [30]. By introducing donor DNA templates, HDR can be used to knock in specific genes at CRISPR cleavage sites [31]. Given this, it has been observed that the Cas9 family targeting RNA. In parallel, diversified RNA-targeted Cas9 systems have been established and opened up new applications [32]. In this study, we focus on reviewing the impact of CRISPR-induced DSBs (knockout or knockin) on cancer immunotherapy.

## A powerful CRISPR toolbox

**Fig. 1:**
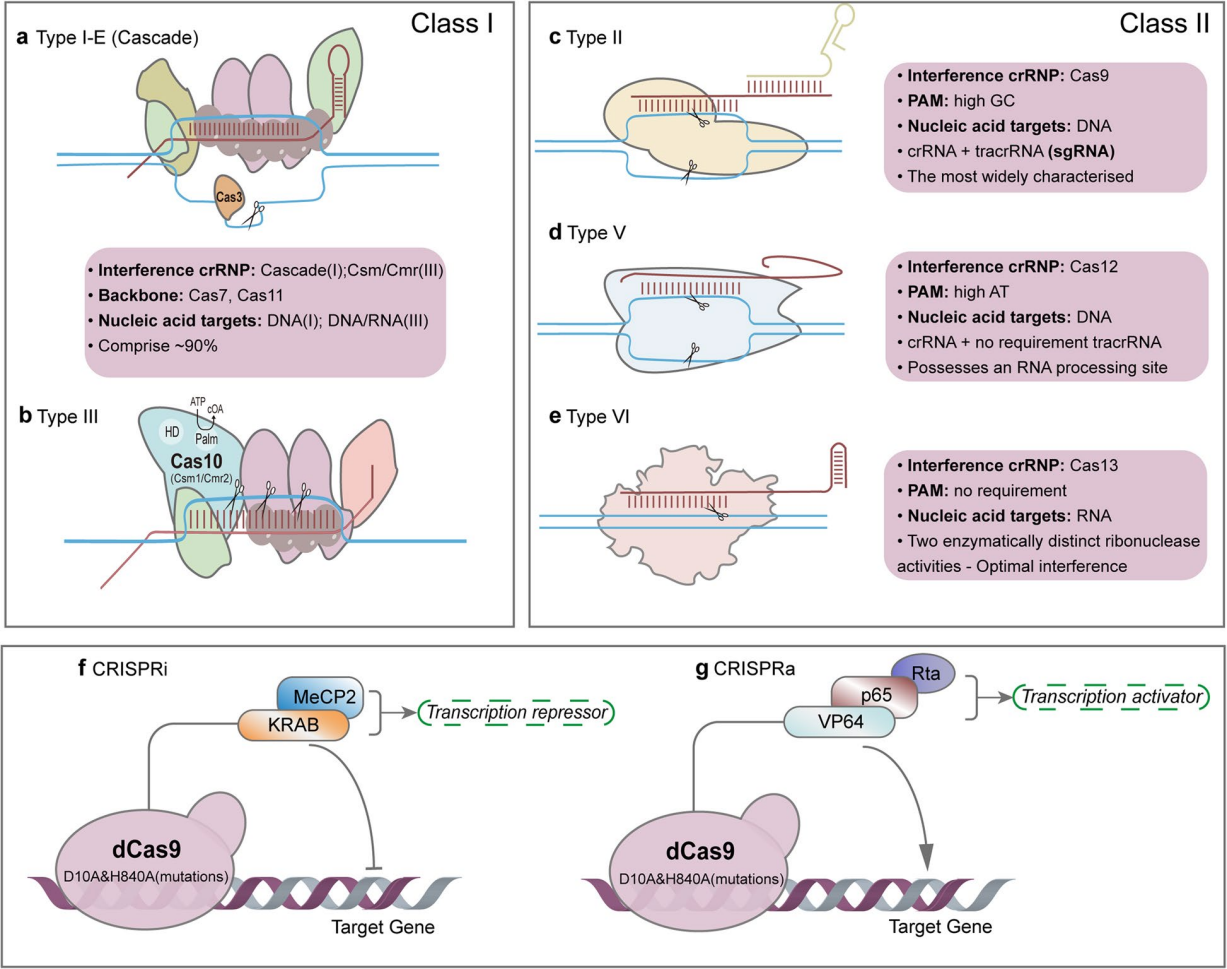
Naturally occurring Cas proteins and the engineered Cas proteins. These systems are divided into two categories: Class I utilize multiple Cas proteins to form effector complexes while Class II perform targeting and nuclease activity with a single Cas protein. **a** Type I-E, also known as Cascade, is a DNA nuclease. **b** Type III, is a DNA/RNA nuclease. The activation of both the HD and the Palm domains of the Cas10 subunit is crucial to confer immunity. **c** Type II (Cas9), has high GC protospacer adjacent motifs (PAM). TracrRNA:crRNA is usually designed as a single RNA complex (sgRNA). Cas9 is the most widely characterised protein. **d** Type V (Cas12), has high AT PAM, can process its own crRNA and possess an RNA processing site. **e** Type VI (Cas13), has no PAM requirement, targets RNA specifically. The Cas9 protein loses cleavage activity by introducing D10A and H840A mutations into the RuvC and HNH domains respectively. **f**, **g** CRISPR activation (CRISPRa) and CRISPR interference (CRISPRi), which activated or repressed target genes by recruiting various effector domains, resulting in transient transcriptional and epigenetic modulation

In addition to editing, the CRISPR-Cas9 system can also regulate gene function by nuclease-deactivated Cas9 (dCas9)-sgRNA complex - CRISPR activation (CRISPRa) and CRISPR interference (CRISPRi), which activated or repressed target genes by recruiting various effector domains, resulting in transient transcriptional and epigenetic modulation [33]. Kira S. et al. [34, 35] summarized the evolutionary classification of the CRISPR-Cas system in 2015 and 2020, respectively. The CRISPR system has two distinct classes, class I and class II. The class I includes types I, III and IV, are defined by multi-Cas proteins, while the class II includes types II, V, and VI, based on Cas9, Cas12 (Cpf1), and Cas13 effectors, singly. Types I is typically utilized to gene editing by recruiting Cas3 nucleases and cascades, or regulating gene expression by cascades alone [36]. Type III CRISPR-Cmr or -Csm systems can attach to sequence-specific RNA targeting and non-sequence-specific and transcription-dependent DNA targeting, thereby conferring the possibility of gene silencing and genome editing by this system [37]. Compared to two nuclease domains of Cas9, Cas12 contains a single RuvC nuclease domain, which cuts double-stranded (dsDNA) or single-stranded DNA (ssDNA) nonspecifically [38], while Cas13 contains two HEPN domains that cleaves RNA specifically [39]. Since the continuous discovery of naturally occurring Cas proteins and the application of engineered Cas proteins, an appropriate CRISPR tool can be chosen from the broadened toolbox to expand the therapeutic potential (Fig. 1).

## Outstanding role-played by CRISPR-Cas9 Technology in the Cellular Therapy

Cancer immunotherapy aims to activate the immune system in an inhibitory condition due to the TME, restoring its ability to kill tumor cells [40]. Generally speaking, cellular therapy is to extract T cells, NK cells, etc. from patients and develop their antitumor activity in vitro. Furthermore, pre-conditioning chemotherapy is required prior to infusion, and these cells are then injected back into the body, enabling personalized cancer treatment. Compared with traditional chemoradiotherapy and other modalities, cell therapies are less toxic and safer if managed properly [41].

### Application of CRISPRs in CAR-T therapy

#### “Arm” the cells via CRISPRs

For oncology patients, the immune system tends to present poor co-stimulation and high co-suppression in TME. Moreover, T cells generally develop phenotypic and functional states that are difficult to exert antitumor effects, such as poor proliferation and severe apoptosis [42]. Relevant molecules can be knocked in via CRISPR-Cas9, so CAR-T can “arm” itself to improve the effect of immunotherapy.

CD40 ligand (CD154) belongs to the TNF-α gene superfamily. CD40L binds to the cognate receptor CD40, participating in multiple immunological processes [43]. Curran et al. [44, 45] aimed to establish the constitutive expression of CD40L for tumor-targeted CAR-T. CD40L+ CAR-T cells revealed superior antitumor effects compared with the control group. CD40/CD40L interaction in vivo produces a direct cytotoxic effect on CD40-expressing tumor cells and further circumvents tumor immune escape. CD40 induces the anti-apoptotic molecule Bcl-xL through CD40L to activate antigen-presenting cells (DCs) via NF-κB pathway [46]. Subsequently, the pro-inflammatory cytokine IL-12 was developed as a vital molecule and mobilized tumors to recognize endogenous T cells [45]. In addition, Wang et al. [47] also reported that HSV-1-derived CD40L-armed oncolytic therapy by CRISPR-Cas9-based gene editing endues TME with the above immune processes, which provides a lasting endogenous immune response in pancreatic ductal adenocarcinoma mice model [48].

Requiring TCR with antigen presentation signals (first signal) and costimulatory signals (second signal) for effector T cell production, cytokines are also irreplaceable (third signal). Editing constitutive expression of interleukin-12 (IL-12) in CAR-T cells with CRISPR-Cas9 may effectively attract macrophages to disrupt TNF-α-mediated processes that result in antigen-losing of tumor cells. Immunosuppressed macrophages and myeloid-derived suppressor cells (MDSCs) lose their inhibitory ability when exposed to IL-12-secreting CAR-T cells [49]. Moreover, IL-12 indirectly mediates innate and adaptive immune processes (as a “bystander”) by improving the activity of type 1 helper T, cytotoxic T, and NK cells [50–52]. To locally accumulate high levels of IL-12 in solid tumor lesions, CAR-T cells were retrovirally transduced with the inducible IL-12 expression cassettes [49]. Therefore, IL-12 can be loaded and transported via CAR-T, like a “cargo”. Correspondingly, IL-15 appears to be associated with T-memory stem cells (TSCMs) [53]. Researchers constructed a chimera co-expressing anti-CD19 CAR and membrane-bound IL-15 (mbIL15) and found that mbIL15-CAR-T cells established the stat5 signaling. The stat5 signaling is capable of inhibiting activation-induced cell death (AICD) [54], strengthening the antitumor activity of CAR-T in vivo [55], and reversing T cell energy [56]. Cytokines discussed above are usually stimulated by the viral construction of expression vectors (γ-virus and lentivirus), but their integration into non-target gene targets has always been a concern. Moreover, overexpression of cytokines may lead to side effects such as abnormal proliferation or toxicity of T cells. Therefore, knocking in specific gene loci (such as T-cell receptor α constant (TRAC)) via CRISPR-Cas9 to properly express these cytokines under the control of endogenous promoters is urgent. Since the TRAC promoter is constitutively active in T cells, under the control of endogenous promoters contributes to more uniform CAR expression in T cells, increases T cell potential, and reduces terminal differentiation and exhaustion [57].

Coincidentally, high expression levels of CXCR2 ligands, such as CXCL1, and CXCL2, have been found to be associated with the growth, invasion, and metastasis of hepatocellular carcinoma (HCC). Jin et al. [58] introduced CXCR2 into HCC-targeting CAR-T cells, indicating that CXCR2 expression can stimulate the “cohesion” of CAR-T cells at the tumor site and ensure their migratory effect to the TME in HCC. Most of the chemokine receptors mentioned above are introduced by viral transduction, and has been shown to be successful for CAR-NK cells, offering homing and tumor infiltration in the TME [59]. Notably, the application of CRISPR-Cas9 technology to express tumor-specific ligands for appropriate chemokine receptor-targeted engineered CAR-T cells holds great promise, although no studies have yet proven the efficacy. Low efficiency of targeted integration of CRISPR/Cas9-mediated knock in is a major factor [60], which may be improved by designing homologous arms of HDRs and screening highly functional sgRNAs [61, 62].

Another “weapon” is the PD1/CD28 chimeric switch receptor (CSR), which converts the inhibitory signal of PD-1 from CAR-T cells to the activation signal of CD28 [63]. Expression of the extracellular domain of PD-1 also competitively binds to PD-L1 in tumor cell surface receptors [64]. A reduction in the incidence of CRS for CAR-T can be found after CRS is armed. Although no studies have yet demonstrated the effects of using CRISPR to knock CSR into CAR-T, the functions of CSR will provide a new line of research with us.

#### Knock out immune checkpoints, eliminating “immune brakes”

TME harbors a variety of immunosuppressive cells, such as MDSCs, tumor-associated macrophages (TAMs), and regulatory T cells (Tregs). Tumor cells up-regulate ligands (e.g., PD-L1) and produce other common inhibitory signals, generating a “brake” effect. In this condition, CAR-T cell therapy can be developed by knocking out these molecules using CRISPR-Cas9.

**Fig. 2:**
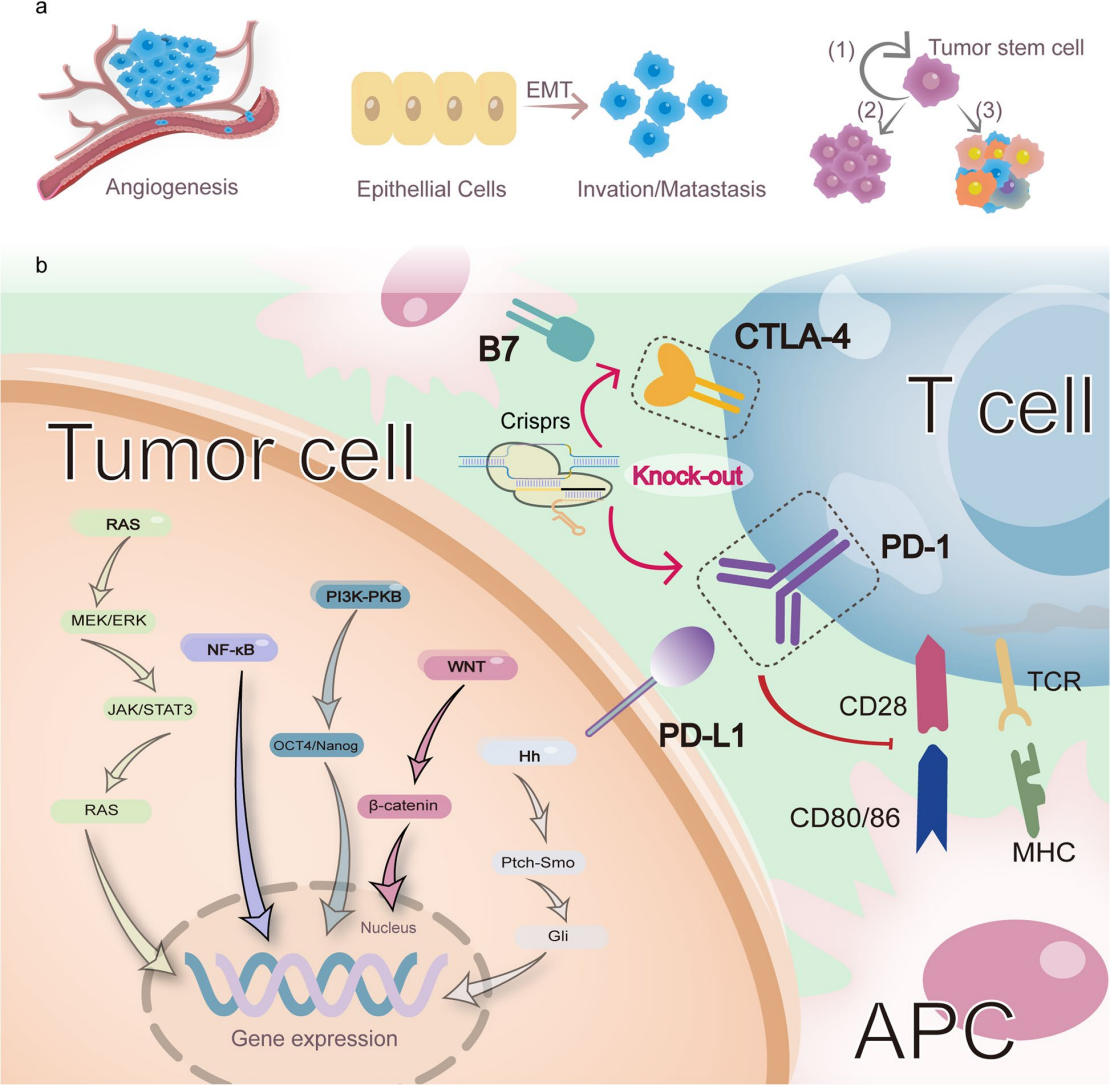
Role of PD-1/PD-L1 axis in tumor progression and utilization of CRISPR-Cas9 to block the PD-1 and CTLA-4 in combination. **a** When PD1−/PD-L1 binding, downstream signaling brings about tumor gene expression as angiogenesis (offer tumor nutrition and promote metastasis), EMT phenomenon (decrease adhesion between epithelial cells and develop tumor invasion and metastasis), and accelerating cancer stem cell generation [cancer stem cells possess (1) self-renewal; (2) proliferation; (3) differentiation traits]. **b** Tumor cells up-regulate the PD-L1 to activate PD-1 on the surface of T cells, then diverse downstream events such as CD28 signal transmission are terminated and multifarious signaling pathways are activated, such as RAS, NF-κB, PI3K-PKB, WNT, Hh, ultimately giving rise to gene expression that tumor proliferation and survival are developed. Another pivotal immune checkpoint is CTLA-4, binding to the B7 receptor on the antigen-presenting cells, which has a inhibitory role in T-cell function. Hence, utilizing CRISPR-Cas9 to block the PD-1 and CTLA-4 in combination may generate higher antitumor responses in CAR-T cells than knocking out PD-1 alone

Initially, the research focus is programmed cell death protein 1 (PD-1) [65], a checkpoint inhibitor presented on the surface of activated T cells that can bind to the PD-L1 receptor on tumor cells. The expression of PD-L1 can be promoted by different signaling pathways, including genetic changes of PD-L1 (such as gene amplification or transcription disorders) and epigenetic mechanisms (such as the dis-proportionality of microRNAs, abnormal DNA histone, and methylation modification). Binding of PD-1 to PD-L1 can cause phosphorylation of two tyrosines in the cytoplasmic domain of PD-1; phosphorylated PD-1 directly or indirectly recruits the cytoplasmic tyrosine phosphatases Shp2 and Shp1 [66, 67], thereby activating tumor proliferation and survival by terminating various downstream events such as CD28 signaling (Fig. 2).

The Su group [68] described a non-virus-mediated method that demonstrated the possibility of electroporation to reprogram T cells by knocking out PD-1 from plasmids encoding sgRNA and Cas9. In addition, PD-L1 may be deleted using indirect methods. Tu et al. performed CRISPR-Cas9 encapsulated in nanoparticles to specifically knock out cyclin-dependent kinase 5 (Cdk5) gene [69], thereby significantly reducing PD-L1 expression in tumor cells. Enhanced CAR-T cytotoxicity was shown to CRISPR-Cas9-mediated PD-1 depletion above. Moreover, another pivotal immune checkpoint is CTLA-4. Hence, utilizing CRISPR-Cas9 to block the PD-1 and CTLA-4 in combination may generate higher antitumor responses in CAR-T cells than knocking out PD-1 alone (Fig. 2) [70].

In addition to the aforementioned immune checkpoints, certain metabolic regulators, transcription factors, and signaling molecules develop multidimensional immunosuppressive signaling networks that can also lead to T cell dysfunction. Firstly, the T cell immunoglobulin domain and mucin domain-3 (Tim-3) is an inhibitory molecule mainly expressed by activated T cells [71]. The Tim-3/galectin-9 pathway promotes tumorigenesis and progression [72], directly leading to Th1 cell death or promoting the role of MDSCs in TME. Subsequently, another negative regulator of T cells is lymphocyte activation gene-3 (LAG-3). LAG-3 is a crucial inhibitory receptor that competes with CD4 for binding to MHC-II [73]. Moreover, LAG-3 inhibits T cell functions by generating Treg cells [74]. Zhang et al. [75] successfully obtained the LAG-3 knockout CAR-T cells using CRISPR-Cas9-mediated gene-editing technology, which strengthened T cell response and facilitated cytokine production. Deletion of diacylglycerol kinase (DGK) in CAR-T cells [76] stimulates CD3 signaling and increases resistance to the immunosuppressive factors TGF-β and prostaglandin E2. Hence, preventing diacylglycerol from interacting with essential proteins in CD3 signaling, such as protein kinase C (PKC) and Ras guanyl releasing protein 1 (RasGRP1), can maintain CAR-T activity in TME. DGK has also been shown to affect various tumor pathways [77], particularly mTOR and HIF-1α pathway. Of note, given the lack of specific inhibitors against this kinase, applying CRISPR-Cas9-targeted knock-out DGK is a groundbreaking cancer immunotherapeutic approach [76]. Another knockout target is the Fas ligand, as Fas-FasL-dependent AICD induces apoptosis and impairs the activity of CAR-T cell [78]. It has been demonstrated that the combination of FAS-KO and DGK-KO is an engineering method to further improve the clinical efficacy of CAR-T cells [77]. Likewise, T cell failure is associated with activation of the nuclear factor of activated T-cells (NFAT). The researches indicated that the deficiency of nuclear receptor 4A (NR4A) induced by Cre/LoxP, a site-specific knockout system mediated by Cre recombinases, leads to the down-regulation of PD1 and TIM-3 [79]. Thus, the inhibition of NR4A family members via CRISPR-Cas9 has a broader range than a single blockade, providing us with new candidate targets.

#### Suppress TGF-β-mediated immune escape in TME like a “Guardian”

Transforming growth factor-β1 (TGF-β1) has been confirmed to be a multifunctional cytokine, which not only regulates the proliferation and differentiation of normal cells [80], but also plays an essential role in tumor initiation and metastasis. First of all, TGF-β1/Smads pathway is the classical signaling pathway [81]. Smad signaling has the significance of inhibiting c-myc expression, promoting cyclin P21 and P15 expression, and down-regulating HPVON gene E6 and E7 levels, which can induce cell cycle arrest, apoptotic response, and senescence [82, 83]. TGF-β1 is also involved in other signaling pathways, collectively referred to as Smad-independent pathways [84], including mitogen-activated protein kinase (MAPK) signaling and Rho family of small GTPases (Rho-like GTPase) signaling. Generally, these pathways have been implicated in epithelial-mesenchymal transition (EMT), angiogenesis, tumor cell motility, and migration. During tumor progression, loss of tumor proliferation inhibition is due to the hindrance of downstream Smad signaling [85]. Further studies showed that TGF-β1 promoted tumor growth through non-Smad signaling when TGF-β signaling changed from “friend” to “enemy”. CAR-T cells are exhausted because of PD-1 and induced as a Treg-like phenotype depending on FOXP3, which is why CAR-T can escape from tumor cells. Tang et al. [86] knocked out the negative effects of TGFBR2 in CAR-T cells using the CRISPR-Cas9 system, thereby wholly blocking TGF-β signaling. Furthermore, the immunosurveillance function of CAR-T cells is improved in TGF-β1-rich TME, showing durable and rapid proliferation, which provides a new idea for improving CAR-T cell therapy. A soluble Fc:TGF-beta type II receptor fusion protein (Fc:TbetaRII) has been found a beneficial effect in preventing tumor metastasis by binding to the receptor of endogenous TGF-β [87]. SB-431542 is a specific TGF-β receptor kinase inhibitor, which has been shown to inhibit TGFBR1 phosphorylation of Smad [88]. Therefore, Combining CRISPR editing with the coadministration of soluble protein inhibitors and small molecule inhibitors [87, 89, 90] is a vital strategy to overcome the TGF-β-mediated immune escape environment.

#### Increase “engine power”, decrease “accident” occurrence — ensure durability and safety

Although CAR-T cell therapy has achieved outstanding achievements in treating cancers, it often occurs in apoptosis, depletion, and even “self-killing” phenomena, that is, insufficient motility and persistence; or leads to severe adverse reactions. For instance, CRS and neuroinflammation with potential toxicity are of widespread concern, which can prove fatal if not carefully managed.

Lentiviral vector-mediated CAR transgene insertion disrupts the gene encoding the methylcytosine dioxygenase TET2 [91], producing powerful CAR-T cells with short-lived memory cell characteristics, which can expand effector cell responses and show the central memory phenotype (CD62L+ cells) associated with higher antitumor activity in vivo. Notably, conventionally prepared CAR-T cells often employ γ-retroviral vectors to transfer CARs into T cells and random insertions usually occur. Several groups [92, 93] targeted CD19-specific CAR to the TRAC locus via CRISPR-Cas9. A gRNA targeting the 5′ end of the first exon of TRAC is designed, and subsequently applied adeno-associated virus (AAV) vector to deliver the CD19 CAR gene sequence. Those studies has revealed CAR expression was homogeneous and constant. Hence, the anticancer potential of T cells is developed by reducing insertional carcinogenesis, endogenously controlling CAR expression, decreasing constitutive signaling, and delaying T cell failure. Another benefit is that surface expression of endogenous TCR can be eliminated by integrating CAR into the TRAC locus, thereby diminishing the risk of TCR-induced autoimmunity and allogeneic responses.

T-cell malignancies such as T-cell acute lymphoblastic leukemia (T-ALL) are a group of hematologic tumors. However, the co-expression of target antigens by CAR-T cells and malignant tumor T cells gives rise to the phenomenon of “self-killing”. Of note, CRISPR-Cas9 gene editing is engineered to disrupt the surface expression of TRAC and CD7 [94], thus preventing CAR-T cells from being suicidal. Similarly, CRISPR-Cas9 gene editing aiming at CD5 has been observed similar self-killing resistance effects in CAR-T cells [95].

CRS is a severe adverse reaction of CAR-T therapy. T cells and effector cytokines (e.g., IL-2, IFN-γ, monocyte chemoattractant protein-1 [MCP-1]) are activated in large amounts by triggering a positive feedback loop. In parallel, immune cascades and cytokine storms are generated, ultimately leading to other severe toxicities such as fever, vasodilatory shock, and even multiple organ dysfunction [96, 97]. Moreover, GM-CSF has been reported to play a crucial role in the development of CRS, which is chiefly produced by monocytes and macrophages [97]. In principle, the incidences of CRS and inflammation will be decreased by significantly reducing the GM-CSF gene through CRISPR-Cas9. Sterner et al. [96] utilized a gRNA with high-efficiency knockout, which is cloned into a CRISPR lentivirus backbone. Hence, diminishing the occurrence of accidents via CRISPR-Cas9 ensures CAR-T cell therapy has higher safety and potential.

#### Generate “fashion stars” for cancer immunotherapy — “off-the-shelf” allogeneic CAR-T cells

We are endowed with two types of CAR-T cell therapy according to the source of T cells: autologous or allogeneic. Both FDA-approved CAR-T cell therapies are autologous [98]. However, autologous therapy has a wide range of limitations compared with allogeneic therapy. Based on this, we are delighted to discover that CRISPR-Cas9 can produce “off-the-shelf” universal allogeneic CAR-T cells. Relative to autologous, “off-the-shelf” allogeneic CAR-T cells have many advantages [99], including 1) reducing cost and time, which can immediately generate CAR-T for patients with rapidly progressive disease (such as aggressive malignancies); 2) ensuring high-quality, an adequate number of T cells, breaking the production constraints of lymphopenia, with controllability; 3) relatively decreasing heterogeneity, realizing standardized CAR-T production and efficient treatment of multiple patients.

**Fig. 3:**
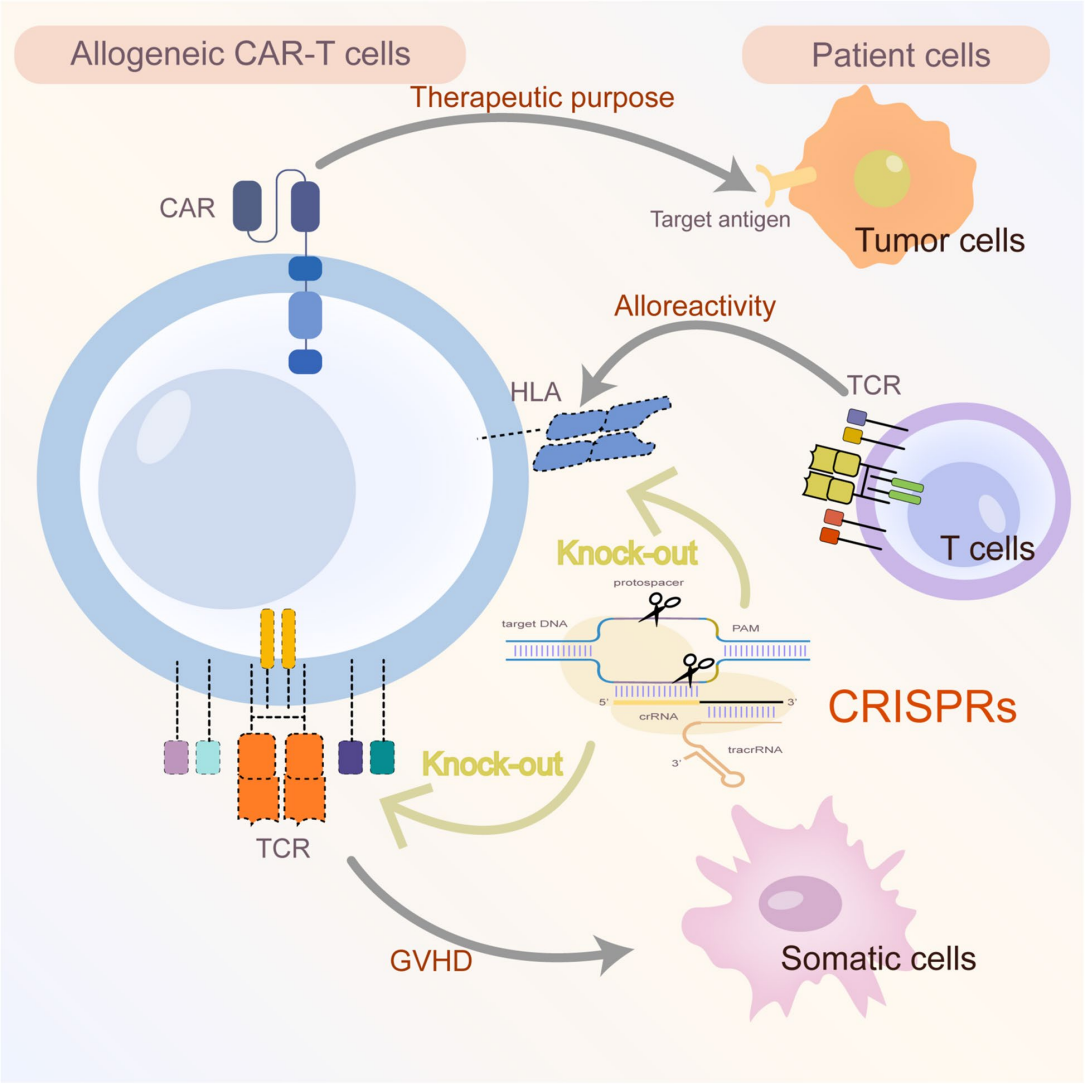
Generate “off-the-shelf” allogeneic CAR-T cells via CRISPRs. Given the presence of endogenous TCR and HLA on donor T lymphocytes, the most significant challenge with universal products is the potential risk of Graft-Versus-Host-Disease (GVHD) and alloreactivity (host versus graft response). GVHD is caused by targeting patient somatic cells mediated by donor T cell TCR-αβ receptors, resulting in an allogeneic T cell attack. Conversely, alloreactivity occurs when patient T cell TCR-αβ receptors recognize exogenous HLA molecules on donor T cells, giving rise to rapid rejection. CAR-T capacity and safety can be enhanced by CRISPR-Cas9 which efficiently knocks out multiple genetic loci with a single pass

Given the presence of endogenous TCR and HLA on donor T lymphocytes, the most significant challenge with universal products is the potential risk of Graft-Versus-Host-Disease (GVHD) and alloreactivity (host versus graft response) [98] (Fig. 3). GVHD is caused by targeting patient somatic cells mediated by donor T cell TCR-αβ receptors, resulting in an allogeneic T cell attack. Conversely, alloreactivity occurs when patient T cell TCR-αβ receptors recognize exogenous HLA molecules on donor T cells, giving rise to rapid rejection. In contrast to ZFNs and TALENs [100, 101], CRISPR-Cas9 can efficiently knock out multiple genetic loci with a single pass. TCR- HLA- Class I, Fas- TCR- HLA- Class I, PD1- TCR- HLA- Class I universal CAR-T cells are produced easily by Ren et al. utilizing CRISPRs. In addition, β2 microglobulin (B2M) is also confirmed to be necessary for HLA-I heterodimer expression on the cell surface [102]. Liu and colleagues generated CAR-T cells with three-target knockouts targeting B2M, TRAC, and PD-1 [103]. In those CRISPR-mediated multiple KO studies, sgRNAs targeting the first exon of B2M, TRAC, PD-1 and other molecules were designed, Cas9 protein and in vitro-transcribed sgRNA were mixed, and gene-disrupted CAR-T cells were generated by combing the lentiviral delivery of CAR with the electro-transfer of CRISPR/gRNAs. CB-010 is a first-in-human, Phase 1, CRISPR-edited allogeneic anti-CD19 CAR-T cell therapy in patients with relapsed/refractory B cell non-Hodgkin lymphoma. Surprisingly, compared with conventional allogeneic anti-CD19 CAR-T cells, the strategy is designed to increase therapeutic indexes by PD-1 gene knockout, site-specific insertion of CAR into TRAC locus and Cas9 CRISPR hybrid RNA-DNA editing (NCT04637763). Conceivably, universal allogeneic CAR-T cells generated by the multiplex genome editing capabilities of CRISPR-Cas9 are more efficient and safer than unmodified cells in tumor cytotoxicity. Given the resources, cost, and time invested, the production of autologous T cells remains the bottleneck for the large-scale clinical application of CAR-T therapy. Unfortunately, some issues of the CRISPR-multiple edits remain to be addressed, such as rearrangement-driven chromosomal abnormalities, genotoxicity, and decreased cellular fitness [104].

#### Other cellular immunotherapies via T cells

TCR-β and TCR-α pair encoded by a DNA cassette recognizing NY-ESO-1 antigen was also introduced into the first exon of TRAC locus, RNPs (complex of gRNA with Cas9) and HDR templates were electroporated following initial T cell stimulation, which is TCR-T therapy [105]. Precise TCR knock-in into specific genetic loci by CRISPR-Cas9 can offer more therapeutic potential to clinics. Nevertheless, TCR-T cell therapy remains problematic. Initially, mismatches between TCR transduced via TCR-T and endogenous TCR subunits (e.g., α and β chains) result in the formation of mixed TCR dimers that form unpredictable epitope specificity. Subsequently, TCR-T cells lack the ability to target antigens, or recognize self-antigens and MHC to elicit autoimmune responses [101, 106]. Next, endogenous TCRs contend with transduced TCRs for the CD3 [107]. Consequently, genes encoding constant regions of the α chain (TRAC) and β chain (TRBC) can be targeted by CRISPR-Cas9 [108, 109], thereby decreasing the probability of adverse effects. Legut and Roth et al. [105, 109] demonstrated improved expression and function of the transgenic TCR due to deletion of the endogenous TCR compared to unmodified. To construct and validate CRISPR-mediated TCR-β constant regions knockout, gRNAs targeting the first exon of the TRBC gene segments were designed, and the T cells were transduced and activated with lentiviral particles.

Tumor-infiltrating lymphocytes (TILs) have been proved to be efficient in treating metastatic melanoma. To further improve the curative effect of TIL therapy, CRISPR-Cas9 can be designed to reverse the repressed state of T cells. For instance, the metabolism-related factor Regnase-I is a negative regulator of antitumor response [110]. Hence, knocking down Regnase-I allows much better infiltration and persistence of T cells in TME. Another research confirmed that the zinc finger transcription factor Gata-3 is non-negligible, which leads to the dysfunction of T cell. Singer et al. [111] transduced sgRNAs targeting Gata-3 along with lentiviruses CRISPR-Cas9 into CD8 + T cells, demonstrating that the antitumor function of TILs can be progressed with increased frequency of IFNγ+ and IL-2+ cells on account of the disruption of Gata-3.

### CRISPRs point to future directions for other cellular therapies

As crucial components of innate immunity, NK cells and macrophages are the first-line of defense. NK cell-mediated killing is not antigen-specific, and NK cells fail to cause GVHD commonly seen in allogeneic T cells (HLA matching) [112], making them ideal candidates for off-the-shelf cell therapy products. Therefore, MHC-I-negative tumor cells can be eliminated by NK cells independently. However, NK cells may become functionally exhausted in TME. CRISPR/Cas9 genome-editing technologies have been applied to improve the issue [113].

Critical understanding of multiple modes of anergy, exhaustion, and senescence, such as reduction of effector cytokines or impairment of cytotoxicity, presence of inhibitory cytokines, regulatory immune cells, and dysregulated receptor signaling found in TME [114], will guide design patterns to enhance NK cell functions. In accordance with these, reactivation of NK cells engineering with CRISPR-Cas9 is an appreciable immunotherapeutic approach. CRISPR/Cas9 genome editing was applied to disrupt CD38 during amplification, showing that CD38 CAR-NK cell fratricide reduced and augmented abilities to target primary acute myeloblastic leukemia (AML) blasts [115]. Velasquez et al. [116*]* have first generated CAR-NK cells engineered using CRISPR-Cas9 targeting CD22 and simultaneously redirecting bystander T cells for CD19 targeting B-cell malignancies to enhance antitumor effects and prevent immune escape. The two-pronged cell therapy presents a promising addition in gene editing of cancer immunotherapy for B-cell malignancies. The effector function of NK cells can also be enhanced by activating receptors. For example, transcriptional activation of MICA - NKG2D ligands show efficient anti-pathogenic cell immunity with an engineered CRISPR-Cas9 system [117]. Like CAR-T immunotherapy, it is desirable to express specific chemokine receptors on the surface of NK cells by CRISPR-Cas9. The CCL19/21-CCR7 axis can be used to promote NK cell infiltration, and chemokine receptor CCR7-bearing NK cells genetically reprogrammed by cGMP-compliant mRNA electroporation method showed enhanced migratory ability towards their ligands CCL-19 and CCL-21 and offered tumor infiltration [118]. Furthermore, using the CRISPR-Cas9 system to genetically disrupt inhibitory pathways and improve immune checkpoint blockade opens a new window in improving the effector functions of NK cells. Blockade of inhibitory NKG2A receptor promotes effector function of NK cells in adoptive therapy [119]. Pomeroy et al. [120] programmed CRISPR-Cas9 to achieve high-efficiency knockout of the NK inhibitory signaling molecules ADAM17 and PD-1 genes, and revealed that NK cell antibody-dependent cytotoxicity was strengthened. Zhu and Daher et al. [121, 122] respectively knocked out a vital cytokine checkpoint (cytokine-induced SH2-containing protein [CISH or CIS]), which is a key negative regulator of IL-15 signaling in NK cells. Meanwhile, TIGIT and CD96 have also been demonstrated to be inhibitors of NK cell activity [123]. CD96 and NKG2A were knocked out simultaneously, with high double KO efficiency [124]. Other emerging human NK cell checkpoints, including LAG3, CISH, TIM3, and signal-regulatory protein (SIRP) α-CD47 [125, 126], are also being progressively explored in the field of cancer immunotherapy. Disruption of Smad3, a downstream mediator of TGF-β signaling, can increase IFN-γ and granzyme B in tumors as well as enhance the antitumor activity of NK cells [127]. Overall, CRISPR-based approaches to knock out the above pathways can revitalize their cytotoxicity and antitumor abilities.

Cancer cells overexpress CD47, which is involved in the SIRPα-CD47 pathway, on their surface. Notably, this pathway is a phagocytic checkpoint for macrophages. When the pathway is activated, the “do not eat me” signal to macrophages is transmitted [128]. Thus, CRISPRs knockdown of SIRP-α in macrophages blocks immune escape, enhancing antitumor effects [129]. Moreover, through pooled in vivo CRISPR knockout (CRISPRko) screens, Wang et al. [130] identified the E3 ligase Cop1 as a modulator of macrophage infiltration and cancer immunotherapy target.

## Find new targets for cancer therapy using CRISPR-Cas9-based genome-wide screening

CRISPR-Cas9-based unbiased genome-wide T cell screening has been employed to identify genes in mammalian cells that significantly affect cancer cell survival, proliferation, migration, and drug resistance, based on which we can explore new targets for cancer therapy [131]. Compared with RNAi-mediated loss-of-function screening, CRISPR-Cas9 has been regarded as the first choice for genetic screening owing to higher screening sensitivity, smaller off-target effects, and less non-targeted interference [132]. Furthermore, the technology of CRISPR can achieve both loss-of-function (CRISPRko, CRISPRi) and gain-of-function (CRISPRa) that researchers can use for pooled screens [133]. CRISPR genomic screens for human T-cell-based therapies have been used to uncover target genes, including key signaling pathways that regulate T-cell effector functions. Manguso et al. [134] identified protein tyrosine phosphatase non-receptor type 2 (PTPN2) as a novel cancer immunotherapeutic target by performing a pooled CRISPR knockout screen in vivo. A sgRNA library targeting 2398 genes genome-wide was transduced into B16 tumor cells, and the top expressed genes were selected by using RNA-sequencing (RNA-seq) to identify a cancer immunotherapy target. PTPN2 has been confirmed as a phosphatase involved in signaling processes, which is mediated through IFN-γ sensing. Antigen presentation and antitumor toxicity of T cells were increased by PTPN2 deletion [130]. Dong et al. [135] performed genome-scale CRISPR screens in CD8 T cells by constructing MKO library and discovered a regulators of tumor infiltration for DHX37 in modulating NF-κB. CRISPRko usually makes the signal clearer, but it tends to be irreversible compared with CRISPRi. Chen et al. [136] construed a in vivo CRISPR screening platform with an optimized retroviral based-sgRNA expression strategy to identify Fli1 as a key transcription factors that regulates effector CD8+ T cell differentiation. Meanwhile, an in vitro T cell exhaustion assay compatible with genome-wide CRISPR screening enabled Belk et al. to identify chromatin and nucleosome remodeling factors, including the cBAF and INO80 complexes that limit T cell persistence [137]. Another finding performed a genome-wide CRISPR loss-of-function screen, revealing ncBAF as a pivotal target for suppressing Treg cell function, which played a foremost role in TME decreasing curative effect of cancer immunotherapy [138]. Ye et al. [139] developed a CRISPRa screen and reported that PRODH2 reprogramming enhances CAR-T cell therapy by developing broad gene expression and metabolic programs. Lymphotoxin-β receptor was identified as a synthetic driver of T cell proliferation through the NF-κB pathway by performing a genome-scale CRISPRa screen, using a lentiviral library of barcoded human open reading frames [140]. Meanwhile, CRISPRa and CRISPRi are required for the comprehensive discovery of functional cytokine regulators. Schmidt et al. [141] identified gene networks controlling IL-2 and IFN-γ production in primary human T cells using paired CRISPRa and CRISPRi screens. Importantly, they were found to be non-toxic and specificity, which facilitated the widespread applications of pooled genome-scale screening. However, more attention should be paid to conditional false positives arising from dropout screening for aneuploidy cancers [142]. CRISPR screening combined with single-cell RNA-sequencing is a powerful method to facilitate high-throughput functional analysis of complex regulatory mechanisms and heterogeneous cell populations, directly linking gRNA expression to transcriptome responses [143]. EMT is a process in which epithelial cells acquire mesenchymal phenotype, which is associated with tumor progression and resistance to therapy [144]. Figueroa et al. [145] integrated single-cell trajectory analysis with CRISPRi screening to identify receptors and transcription factors facilitating progress along the TME, including regulators of KRAS. Moreover, Perturb-seq has been constructed which is combinatorial CRISPR screens with RNA-seq readout. Dixit et al. [146] inferred gene function by demonstrating Perturb-seq, focusing on transcription factors regulating the response of dendritic cells to lipopolysaccharide. CRISPR-Cas9-based genome-wide screening dramatically increases the scope of of cancer therapy.

## Excavate potential of spatial CRISPR genomics

Combining CRISPR technology with single-cell RNA sequencing, a group of sgRNAs are introduced into cells, or barcoding the protein, and they can be detected [147]. Dhainaut et al. [148] developed a spatial functional genomics platform termed Perturb-map combines CRISPR pools, multiplex imaging, and spatial transcriptomics to address the role of perturbation genes in lung tumors by analyzing the molecular status of tumor lesions when different genes are knocked out. Spatial CRISPR screening confirmed tumor-facilitating effects of immune checkpoints (PD-L1 and CD47), and also identifies tumor composition, organization, and immune infiltration in the TME, following Socs1 and TGFBR2 KO lesion. CRISPR-GO system is developed to provide a programmable and versatile platform for the localization of targeted genomic DNA in the nucleus, leading to a deeper understanding of spatial genome organization [149]. To minimize genotoxicity in editing, spatialization and temporalization of CRISPR process is desirable. Local magnetic spatial activation of MNP-BV-CRISPR nanoparticles can achieve specific gene modifications [150]. Spatial and temporal control of gene editing using liposome vectors reported by Yagiz et at [151]. will open up a new avenue for wider CRISPR-Cas9 gene-editing translation. Moreover, chemical and Optogenetics (paCas9) induction have been used to permit temporal control of CRISPR-mediated multiple genes targeting [152].

## Current applications of CRISPR-Cas9 Technology in Basic Research and Translational Medicine

CRISPR-Cas9 technology has been widely applied in basic research and translational medicine, which are the solid foundation of science research. Reproduce cancer-related events by producing cancer models is essential and the production of CRISPR-mediated-KO mice has become routine practice. By delivering combinations of sgRNAs targeting five genes and Cas9 with a lentiviral vector, Heckl et al. [153] induced the development of AML in a single mouse hematopoietic stem cell. A study have demonstrated that loss of p107 and p130 significantly facilitated tumor progress in small cell lung cancer by Trp53/Rb1 double CRISPR-knockout model [154]. Patient-derived xenograft (PDX) animal models achieve the exogenous growth of human tumors and provide an preclinical tool for oncology research. He et al. [155] knocked out Rag1, Rag2, and Il2rg in Sprague Dawley rats and successfully developed a PDX model of lung squamous cell carcinoma, holding great potential to serve as a new model for oncology research. In addition to KO models, base editing by co-injection of Cas9 mRNA and sgRNA into one-cell stage embryos also has great potential in constructing animal models and modifying mutant genes [156]. Organoids are the advanced in vitro 3D cell cultures and can be genetically coded utilizing CRISPR-Cas9 to explore new therapy and identify gene function. Cas9 and lentiviruses expressing sgRNAs targeting four breast cancer-associated tumor suppressor genes sequential introduced into normal organoids was used to mimic neoplasia, which is helpful to explore the pathogenesis of breast cancer [157]. Distinct pathways downstream of oncogenic ARID1A mutation were defined by ARID1A knockout in primary human gastric organoids. Another important application of CRISPR in cancer research is tracing evolution dynamics in tumours. Bowling et al. [158] presented the CRISPR array repair lineage tracing (CARLIN) mouse system that can be used to interrogate the lineage information of single cells, which is inducible, transcribed barcodes, and over time. Moreover, a system was described achieving labeling genomic loci in living cells with dCas9 and engineered sgRNAs known as the CRISPRainbow. The sgRNAs each recruited a pair of fluorescent proteins that were fused to RNA hairpin, enabling scientists to simultaneously regulate or label multiple loci to observe the entire process of the gene regulatory network [159]. Using CRISPRs-based diagnostic system for detecting cancer is another robust application. Gootenberg et al. [160] described an in vitro nucleic acid detection platform called SHERLOCK (Specific High Sensitivity Enzymatic Reporter UnLOCKing), consisting of the RNA-guided RNase Cas 13a and a reporter signal. Another method termed DNA endonuclease-targeted CRISPR trans reporter (DETECTR), enables rapid and specific viral diagnostic platform for molecular diagnostics, consisting of Cas12a and recombinase polymerase amplification [38]. Park et al. [161] designed sgRNAs targeting the endogenous EGFR genomic region flanking the exon encoding T790 to generate a PC9 lung cancer cell line harboring EGFR T790M, investigating the molecular mechanisms of tyrosine kinase inhibitors resistance. Shen et al. [162] targeted all pairs of 73 cancer genes with dual-gRNAs in HeLa, A549 and 293 T cell lines, altogether comprising 141,912 tests of interaction. The combinatorial CRISPR-Cas9 screens will pay the way for mapping the genetic interaction networks and promote the developments of new druggable synthetic-lethal interactions. Therefore, it is foreseeable that CRISPR technologies could serve as a robust tool for drugs clinical application and development.

## Challenges and prospects

**Fig. 4:**
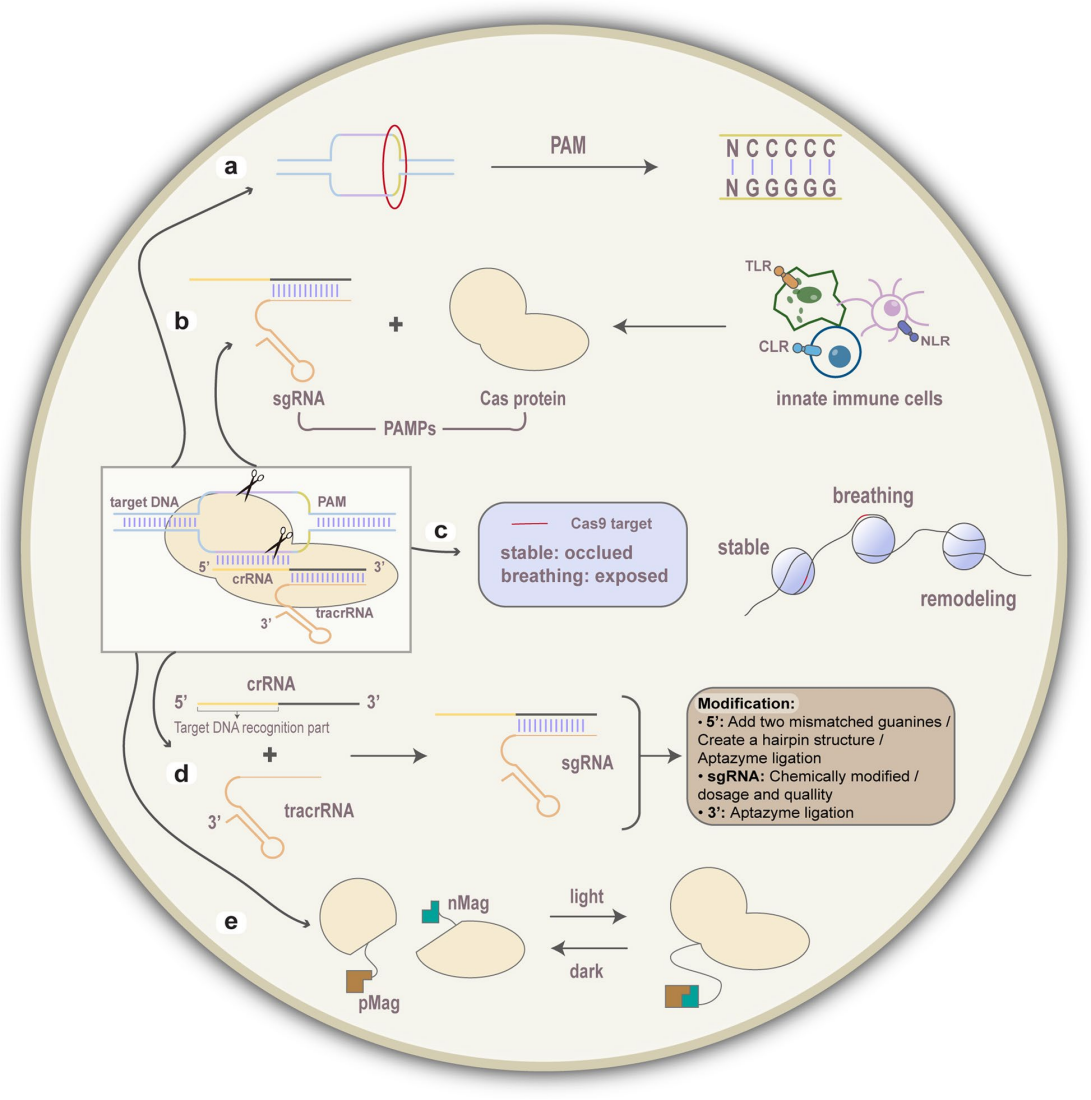
A multitude of factors and regulations may influence gene editing. **a** Higher CG content stabilizes the hybrid and facilitates the efficiency of the Cas9. **b** gRNA and Cas protein are PAMPs for the innate immune cells, and the immune response may affect the results of gene editing. **c** Nucleosome breathing and remodeling have already been investigated that may enhance Cas9 activity. **d** Modification of sgRNAs responsible for target DNA recognition can affect the specificity of Cas9 cleavage. **e** The CRISPR system can be tightly spatially or temporally controlled. Light irradiation induces heterodimerization between pMag and nMag

Although cancer immunotherapy mediated by the CRISPR-Cas9 system has not been widely programmed in clinical practice, clinical trials based on their combination are in full swing (Table 2). In addition to the application of CRISPR-Cas9 introduced above in immunotherapeutic approaches currently under research hotspots, it also plays a cutting-edge role in other immunotherapies such as ameliorating antibody performance [163, 164], changing TME and immune responses (M2/N2/Treg/MDSC) [165–170], and reprogramming MHC specificity (correcting MHC mismatches) [171]. Notably, a multitude of factors and regulations may influence gene editing (Fig. 4). Higher CG content has more hydrogen bonds between sgRNA and target DNA, which stabilizes the hybrid and facilitates the efficiency of the Cas9 [172]. Modification of gRNAs responsible for target DNA recognition can affect the specificity of Cas9 cleavage. The dosage and quallity of gRNA also have impacts on the efficiency and specificity of editing [173]. The CRISPR system can be tightly spatially or temporally controlled, when increasing the effective concentrations of the gRNA-Cas9 complex component and resulting in immediately active complex, with short-lived [174]. After all, gRNA and Cas protein are immunogenic for the immune system, and the immune response may affect the results of gene editing [175]. Meanwhile, DNA end structure and sequence features near the break site and DNA repair mechanisms influence the editing outcome [176]. Genetic variations such as spontaneous mutations and chromosome aberrations compromise the effectiveness of manipulation in the target region [177]. Chromatin accessibility, guide sequence secondary structure, eukaryotic chromatin state, chromosomal rearrangements, DNA methylation, and nucleosome breathing and remodeling have already been investigated that may influence CRISPR-Cas9 editing efficiencies [178–180].

**Table 2:** Application of CRISPR-Cas9 technology in cancer immunotherapy

Condition or diseases	Immunotherapy type	Target sites	Identifier	Phase
T Cell Lymphoma	Allogeneic CRISPR-Cas9-Engineered T Cells (CTX130)	Unknown	NCT04502446	Phase 1
Relapsed/Refractory B Cell Non-Hodgkin Lymphoma	CRISPR-Edited Allogeneic Anti-CD19 CAR-T Cell (CB-010)	Unknown	NCT04637763	Phase 1
Metastatic Non-small Cell Lung Cancer	Autologous lymphocytes	PD-1	NCT02793856	Phase 1
EBV (Epstein-Barr virus) Positive Advanced-Stage Malignancies	EBV-CTL cells	PD-1	NCT03044743	Phase 1/2
Metastatic Non-small Cell Lung Cancer	Engineered T cells	PD-1	NCT02793856	Phase 1
Invasive Bladder Cancer Stage IV	Engineered T cells	PD-1	NCT02863913	Phase 1
Metastatic Renal Cell Carcinoma	Engineered T cells	PD-1	NCT02867332	Phase 1
Hormone Refractory Prostate Cancer	Engineered T cells	PD-1	NCT02867345	Unknown
Esophageal Cancer	Engineered T Cells	PD-1	NCT03081715	Not Applicable
Prostate Cancer	Engineered T cells	PD-1	NCT03525652	Phase 1/2
Advanced Hepatocellular Carcinoma	Engineered T cells	PD-1	NCT04417764	Phase 1
Solid Tumor, Adult	Mesothelin-directed CAR-T cells	PD-1	NCT03747965	Phase 1
Tumors of the Central Nervous System	Unknown	NF1	NCT03332030	Suspended
Human Papillomavirus-Related Malignant Neoplasm	Unknown	HPV E6/E7	NCT03057912	Phase 1
Solid Tumor, Adult	Anti-mesothelin CAR-T cells	Endo-TCR/PD-1	NCT03545815	Phase 1
Multiple Myeloma; Melanoma; Synovial Sarcoma; Myxoid/Round Cell Liposarcoma	NY-ESO-1 TCR-T	Endo-TCR/PD-1	NCT03399448	Phase 1
B Cell Leukemia; B Cell Lymphoma	UCART019	Endo-TCR/B2M	NCT03166878	Phase 1/2
Acute Lymphoblastic Leukemia (ALL) Non Hodgkin Lymphoma (NHL)	UCART019	Endo-TCR/B2M	NCT03229876	Not Applicable
Gastro-Intestinal (GI) Cancer	TIL	CISH	NCT04426669	Phase 1/2
Renal Cell Carcinoma	Allogeneic CRISPR-Cas9-Engineered T Cells (CTX130)	CD70	NCT04438083	Phase 1
Cell Leukemia; U-T-cell Lymphoma	T-CAR-T	CD7/TRAC	NCT04264078	Early Phase 1
High Risk T-cell Malignancies	Non-Edited T Cells (CRIMSON-NE)	CD7	NCT03690011	Phase 1
B Acute Lymphoblastic Leukemia	Allogenic engineered human T cells	CD25/TRAC	NCT04557436	Phase 1
B Cell Leukemia; B Cell Lymphoma	Universal CAR-T Cells	CD19/CD20/CD22	NCT03398967	Phase 1/2
B cell ALL	Allogeneic CRISPR-Cas9-Engineered T Cells (CTX110)	CD19	NCT04035434	Phase 1
Relapsed/Refractory B Cell Non-Hodgkin Lymphoma	Allogeneic CRISPR-Cas9-Engineered T Cells (CB-010)	CD19	NCT04637763	Phase 1
Multiple Myeloma	Allogeneic CRISPR-Cas9-Engineered T Cells (CTX120)	BCMA	NCT04244656	Phase 1

Although CRISPR-Cas9, with its remarkable scalability, flexibility, and operability, provides a powerful technical enablement for achieving the highest target gene editing goal, many obstacles remain. Of note, off-target effects and unsatisfactory delivery systems are significant challenges in curing. Even minimal off-target effects can have unforeseen consequences during clinical treatment. Therefore, it is extremely indispensable to reduce non-targeted effects by selecting proper delivery systems, controlling no or little homologous sequences throughout the genome, avoiding target sequences with high GC content, developing Cas9 variants, applying Cas proteins, and designing sgRNAs with maximum specificity [174, 181–183]. The other barriers for using CRISPR in clinics is the lack of a safe and powerful delivery system to target the tissues and cells. The current systems have been developed to deliver the CRISPR-Cas9 system in vitro and in vivo, but it is difficult to simultaneously achieve all of the anticipated criteria [184]. To address this issue, CRISPR delivery strategies may be continuously improved through the well-studied delivery strategy experience with genes and macromolecules (proteins and nucleic acids) [185]. NHEJ and MMEJ typically causes INDELs of genes to disrupt protein-coding sequences and develops functional knockouts, but may generate deleterious DSB repair byproducts, including cancer-driving chromosomal translocations, large chromatin deletions, and vector insertions in humans. The structural variations (SVs) has become another dimension of threat to genome stability during genome editing [186, 187]. PEM-seq, HTGTS and SuperQ were explored to distinguish various DNA repair products induced by CRISPR–Cas9. The methods provide a channel for further understanding the relevance of SVs to human diseases [188, 189]. Furthermore, immune response against CRISPR system may affect the results of gene editing and even lead to mortality [175]. In a study using adenovirus vector to deliver CRISPR-spCas9, cytokine release and spCas9-specific antibodies were detected, providing evidence that the treatment induced humoral immunity by Cas9 proteins [190]. The CRISPR gRNAs consisting of hairpins are potential pathogen-associated molecular patterns engaging pattern recognition receptors (PRRs) to trigger innate immune responses. By modifying the secondary structure of gRNAs, co-opting gRNA scaffolds less prone to activate innate immunity and altering the localization of CRISPR gRNAs by Pol II or Pol III are elicited to reduce engagement with PRRs [191]. The delivery modality of CpG-depleted AAV vectors could establish more persistent transgene expression by minimizing TLR9 engagement [192]. Co-administering immunosuppressants to decrease immune response is also a recommendation [175]. Moreover, the inefficient repair of DSBs by HDR, low-efficiency delivery of large DNA fragments, and CAR toxicity [193] also need to be addressed in the future.

## Conclusions

So far, immunotherapy stands on the stage of the world’s research centers. In terms of cancer immunotherapy, the toolbox of CRISPR-Cas9 has been continuously expanded through in-depth study. Although the advent of immunotherapy has ushered in a new era in the treatments of solid tumors, it remains limited and requires breaking adverse effects. Meanwhile, abundant solutions with clinical trial supports are urgently required, laying the ground for clinical use of CRISPR-Cas9-modified cancer immunotherapy. Scientists are still needed to make considerable experimental research on the cellular and molecular mechanisms of related targets. These will provide deeper insights into CRISPRs and cancer immunotherapy.

## Data Availability

Not applicable.

## Abbreviations

AAV: Adeno-associated virus
ACT: Adoptive cellular immunotherapy
ADC: Antibody-drug conjugate
AML: Acute myeloblastic leukemia
AICD: Activation-induced cell death
B2M: β2 microglobulin
CAR: Chimeric antigen receptor
CAR-T: Chimeric antigen receptor T-Cell
Cas9: CRISPR-associated protein 9
CRISPR: Clustered regularly interspaced short palindromic repeats
crRNA: CRISPR-derived RNA
CRS: Cytokine release syndrom
CSR: Chimeric switch receptor
dCas9: dead Cas9
DGK: Diacylglycerol kinase
DSBs: Double-strand breaks
EMT: Epithelial-mesenchymal transition
FDA: Food and Drug Administration
gRNA: guide RNA
GVHD: Graft-Versus-Host-Disease
HDR: Homologous recombination repair
INDELs: Insertions and deletions
LAG-3: Lymphocyte activation gene-3
MAPK: Mitogen-activated protein kinase
mbIL15: membrane-bound IL-15
MCP-1: Monocyte chemoattractant protein-1
MDSCs: Myeloid-derived suppressor cells
NFAT: Nuclear factor of activated T-cells
NHEJ: Nonhomologous end-joining
NR4A: Nuclear receptor 4A
PAMs: Protospaceradjacent motifs
PDX: Patient-derived xenograft
PKC: Protein kinase C
PRRs: Pattern recognition receptors
PTPN2: Protein tyrosine phosphatase non-receptor type 2
RasGRP1: Ras guanyl releasing protein 1
Rcas9: RNA-targeted Cas9
Rho-like GTPase: Rho family of small GTPases
RNA-seq: RNA-sequence
SgRNA: Single guide RNA
SIRP: Signal-regulatory protein
SVs: Structural variations
TALENs: Transcription activator-like effector nucleases
T-ALL: T-cell acute lymphoblastic leukemia
TILs: Tumor-infiltrating lymphocytes
TME: Tumor microenvironment
TracrRNA: Transactivating crRNA
Tregs: Regulatory T cells
TSCMs: T-memory stem cells
TRAC: T-cell receptor α constant
ZFNs: Zinc-finger nucleases
